# Imaging Carotid Plaque Burden in Living Mice via Hybrid Semiconducting Polymer Nanoparticles-Based Near-Infrared-II Fluorescence and Magnetic Resonance Imaging

**DOI:** 10.34133/research.0186

**Published:** 2023-07-19

**Authors:** Li Xu, Zhe Li, Yuan Ma, Lingling Lei, Renye Yue, Hui Cao, Shuangyan Huan, Wei Sun, Guosheng Song

**Affiliations:** ^1^ State Key Laboratory for Chemo/ Bio-Sensing and Chemometrics, College of Chemistry and Chemical Engineering, Engineering, Hunan University, Changsha 410082, China.; ^2^ Key Laboratory of Targeted Intervention of Cardiovascular Disease, Collaborative Innovation Center for Cardiovascular Disease Translational Medicine, Nanjing, Medical University.; ^3^ Department of Cardiology, The First Affiliated Hospital of Nanjing Medical University, Nanjing Medical University, Nanjing 210029, Jiangsu, China.

## Abstract

The majority of atherothrombotic events (e.g., cerebral or myocardial infarction) often occur as a result of plaque rupture or erosion in the carotid, and thereby it is urgent to assess plaque vulnerability and predict adverse cerebrovascular events. However, the monitoring evolution from stable plaque into life-threatening high-risk plaque in the slender carotid artery is a great challenge, due to not enough spatial resolution for imaging the carotid artery based on most of reported fluorescent probes. Herein, copolymerizing with the small molecules of acceptor-donor-acceptor-donor-acceptor (A-D-A′-D-A) and the electron-donating units (D′), the screened second near-infrared (NIR-II) nanoprobe presents high quantum yield and good stability, so that it enables to image slender carotid vessel with enough spatial resolution. Encouragingly, NIR-II nanoprobe can effectively target to intraplaque macrophage, meanwhile distinguishing vulnerable plaque in carotid atherosclerosis in living mice. Moreover, the NIR-II nanoprobe can dynamically monitor the fresh bleeding spots in carotid plaque, indicating the increased risk of plaque instability. Besides, magnetic resonance imaging is integrated with NIR-II fluorescence imaging to provide contrast for subtle structure (e.g., narrow lumen and lipid pool), via incorporating ultrasmall superparamagnetic iron oxide into the NIR-II nanoprobe. Thus, such hybrid NIR-II/magnetic resonance imaging multimodal nanoprobe provides an effective tool for assessing carotid plaque burden, selecting high-risk plaque, and imaging intraplaque hemorrhage, which is promising for reducing cerebral/ myocardial infarction-associated morbidity and mortality.

## Introduction

Adverse cardiovascular event is one of the leading causes of death and permanent disability worldwide [1]. Notably, stroke is one of the most frequent manifestations of atherosclerosis and the third leading cause of death after ischemic heart disease and cancer [2]. Approximately 80% of all strokes are ischemic, and embolus arises from carotid atherosclerotic plaques [3]. The damaged plaque can trigger embolism in downstream blood vessels and induce cerebral infarctions [4]. Even though not all carotid atherosclerotic plaques may appear symptoms or lead to stroke, those advanced atherosclerotic lesions are more dangerous [5]. Especially, the stenosis in internal carotid artery is considered to be a higher risk for cerebral ischaemia, when it exceeds 70% narrowing of lumen section [6]. Moreover, the cholesterol deposition [7], inflammation [8], and increased neovascularization [9] play a major role in the rupture of advanced lipid-rich atherosclerotic lesions, which result in an increased risk of cerebrovascular events. Thus, the identification of stable plaque and plaque erosion would be a benefit for reflecting plaque burden and vulnerability in the atherosclerotic lesion, which therefore is important for risk warning, informing clinical treatment, and ultimately developing preventive pharmacotherapy to stabilize plaque and improve patient outcomes [10].

To determine whether patients are required to undergo carotid intervention in current clinical practice is to observe the clinical features combined with the degree of carotid stenosis [11]. However, it is a great challenge to obtain accurate prediction about arteriosclerosis progression, plaque burden, and especially plaque vulnerability, based on conventional angiography and ultrasonography [5]. Currently, medical imaging such as computed tomography, positron emission tomography, and magnetic resonance angiography can be employed to detect the emergence or location of plaques [12]. However, those imaging technologies usually seem helpless to report plaque inflammation, and intraplaque hemorrhage (IPH) (especially for carotid artery) that is regarded as important signals for high-risk plaque and may be prone to cause ischemic stroke and thrombotic complication [13].

Molecule fluorescent probes can utilize biomarkers and metabolites to report and assess disease progression in biological specimens [14]. Recently, various near-infrared fluorophores, such as porphyrin (e.g., Ce 6) [15], cyanine (e.g., indocyanine green [ICG]) [16], Bodipy [17], or aggregation-induced emission [18] based organic dots have been reported for imaging carotid atherosclerotic plaques (Table S1). However, most of those probes were only employed for imaging the excised carotid plaque tissues, because the short wavelength of fluorescence emission (less than 900 nm) and strong autofluorescence of mice hampered the imaging depth in living mice [16,19]. Although several probes were able to image the plaques in vivo, those images still suffered from poor spatial resolution due to the serious photon scattering, which make them difficult to well distinguish plaques in carotid artery in living mice [20]. Thus, it is highly desirable to develop novel imaging technology to assess plaque burden and identify vulnerable plaque in carotid artery, thereby enabling risk stratification to guide clinical decision-making.

Interestingly, second near-infrared (NIR-II, 1,000 to 1,700 nm) fluorescence imaging provides unique advantages such as weak autofluorescence, good spatial resolution, and deeper penetration into biological tissues [21]. Unfortunately, rare NIR-II fluorescent probes have been developed for accurately and dynamically imaging vulnerable plaque in carotid until now. Herein, we designed a series of semiconducting polymers via copolymerizing with the small molecules of acceptor-donor-acceptor-donor-acceptor (A-D-A′-D-A) and different electron-donating-abilities units (D′) including thiophene, benzodithiophene, and dithiophene-substituted benzothiadiazole, respectively. Notably, the absorption and emission of materials could be finely tuned by the introduction of different copolymerization unit to obtain high quantum yields (QYs). Then, we constructed NIR-II fluorescent nanoprobes via nanoprecipitation of various semiconducting polymers. Based on the molecular engineering strategy, NIR-II fluorescent nanoprobes exhibited good fluorescence stability and strong brightness, so that they were able to image vessels with good spatial resolution and high signal-to-background ratio. Due to the good surface modification, NIR-II nanoprobes presented the improved biocompatibility and extended blood-circulation time, which enabled the continuous monitoring of plaque.

Within atheromatous plaques, macrophages are the first group of cells to invade early arterial lesions, thereby transforming into culprit foam cells and forming atheroma [22]. Subsequently, those inflammatory mediators and reactive oxygen species released by neutrophils and lymphocytes play pivotal roles to determine catastrophic thrombotic complications of atherosclerosis (i.e., plaque rupture or erosion and in turn infarction and ischemic stroke) [23]. Considering macrophage as an ideal target for assessing plaque burden, the visualization of intraplaque macrophage via NIR-II fluorescence is expected to be a promising approach [24]. Notably, the as-prepared NIR-II nanoprobe enabled to target inflammatory macrophage within atherosclerotic plaque, presenting the differential signal in carotid artery between healthy mice and mice bearing carotid atherosclerosis or carotid atherosclerosis with acute pneumonia. Furthermore, NIR-II nanoprobes could well image IPH in carotid plaques. Thus, such NIR-II nanoprobe demonstrated the capability for imaging vulnerable atherosclerotic plaques in carotid artery and IPH.

**Fig. 1.**
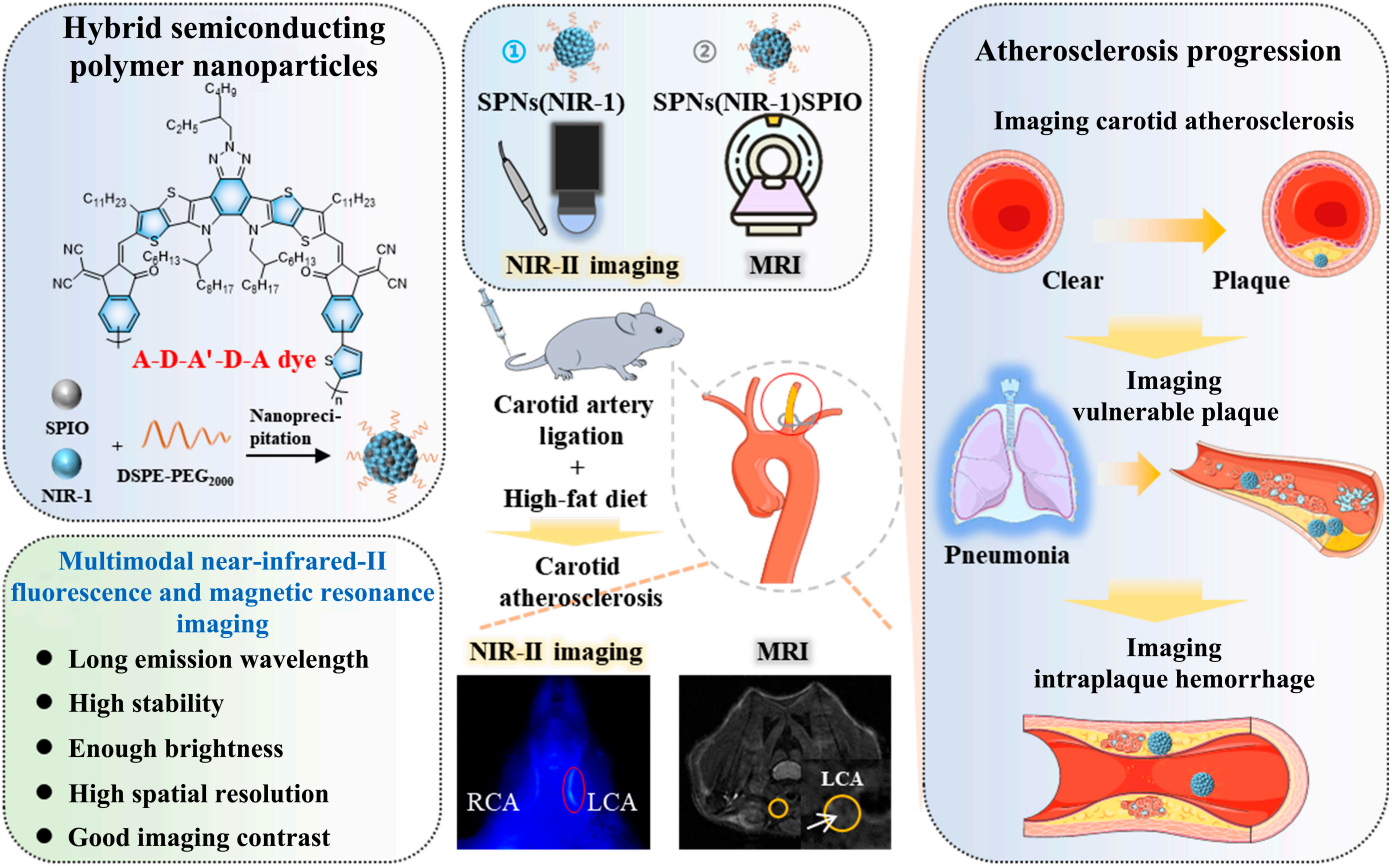
Hybrid SPNs for noninvasive time-lapse imaging of carotid atherosclerosis progression in C57BL/6 male mice via NIR-II fluorescence and MRI, which is promising for assessing and monitoring the high-risk pathological changes within carotid atherosclerotic plaque in living body.

Since magnetic resonance imaging (MRI) can provide excellent complementary information on the vessel lumen and vessel wall [22,24,25], we integrated NIR-II fluorescence imaging with MRI via incorporating ultrasmall superparamagnetic iron oxide (SPIO) into NIR-II fluorescent nanoprobes. Such a hybrid NIR-II/MRI multimodal nanoprobe was capable of identifying plaques in carotid artery. Interestingly, subtle structures within lesion could be well distinguished along with the enrichment of NIR-II/MRI nanoprobes within plaque, including narrow lumen and lipid pool. Thus, the integration of NIR-II fluorescence and MRI presented a high specificity of functional imaging and high resolution of structural imaging in carotid atherosclerosis (Fig. 1).

## Results

### Molecular engineering and synthesis of semiconducting polymers

**Fig. 2.**
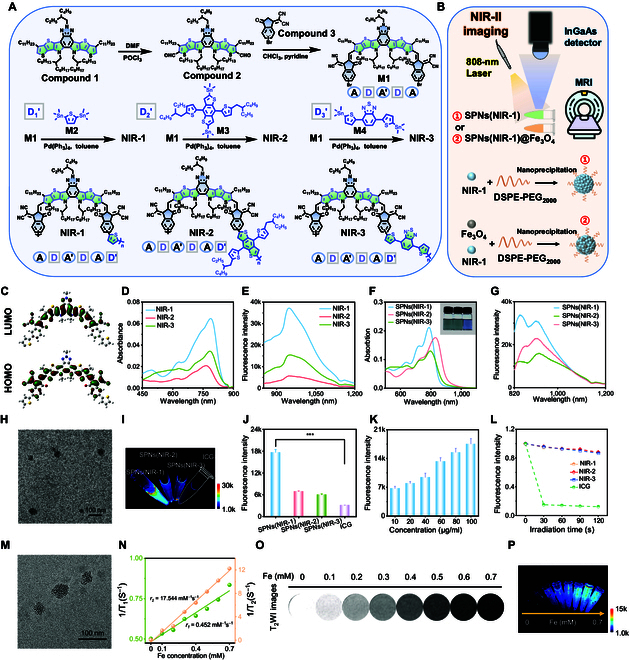
Molecular engineering and synthesis of semiconducting polymers and characteristics of NIR-II fluorescent or NIR-II/MRI hybrid nanoparticles. (A) The synthetic routes for NIR-1, NIR-2, or NIR-3. (B) The preparation of SPNs(NIR-1) and SPNs(NIR-1)@ SPIO and diagram of NIR-II or MR imaging. (C) The density functional theory molecular orbital plots of NIR-1. (D) Absorption spectra of NIR-1, NIR-2, and NIR-3 in toluene. (E) Emission spectra of NIR-1, NIR-2, and NIR-3 in toluene. (F) Absorption spectra of SPNs(NIR-1), SPNs(NIR-2), and SPNs(NIR-3) in water (10 μg/ml). Inset indicated aqueous solutions of SPNs(NIR-1), SPNs(NIR-2), and SPNs(NIR-3). (G) The emission spectra of SPNs(NIR-1), SPNs(NIR-2), and SPNs(NIR-3) in water. (H) Representative TEM image of SPNs(NIR-1). (I) NIR-II images of SPNs(NIR-1), SPNs(NIR-2), SPNs(NIR-3), and ICG (10 μg/ml) under excitation of 808 nm (1 W/cm^2^). (J) The NIR-II fluorescence intensity from (I). (K) Fluorescence intensity of SPNs(NIR-1) under 808-nm laser irradiation (1 W/cm^2^) for different concentrations (10 to 100 μg/ml). (L) The quantized NIR-II fluorescence intensity of SPNs(NIR-1), SPNs(NIR-2), SPNs(NIR-3), and ICG (10 μg/ml) under 808-nm laser irradiation (1 W/cm^2^) for different times. (M) Representative TEM image of SPNs(NIR-1)@SPIO. (N) The *r_1_* (1/T_1_) and *r_2_* (1/T_2_) water proton spin relaxivities of SPNs(NIR-1)@SPIO. (O) T_2_WI images of SPNs(NIR-1)@SPIO at different Fe concentrations (mM) using a 7.0-T MRI scanner. (P) NIR-II fluorescence imaging of SPNs(NIR-1)@SPIO.

We employed the polymerization strategy to extend the π-conjugation of molecules and improve luminance and stability. Firstly, the heptacyclic-fused-ring core (D-A′-D) was conjugated with a strong electron-withdrawing unit (A, dicyanovinylindanone), resulting in acceptor-donor-acceptor-donor-acceptor (A-D-A′-D-A) molecule with intense intramolecular charge transfer (ICT) effect. Then, 3 kinds of semiconducting polymers were synthesized via copolymerization of A-D-A′-D-A molecule and various donor units (i.e., thiophene, benzodithiophene, or dithiophenbenzothiadiazole), namely, NIR-1, NIR-2, and NIR-3 respectively (Fig. 2A). By introducing a copolymerization unit (D′) to form an A-D-A′-D-A-D′ structure, the multiple ICT was realized, narrowing the band gap of those semiconducting polymers (Fig. S1 and Table S2).

The synthetic routes and chemical structures of those semiconducting polymers were depicted in Fig. 2A. The synthesis of polymers began with compound 1. The aldehyde group was introduced into the α-position of the thiophene unit by the Vilsmeier–Haack reaction to give compound 2. Aromatic aldehydes reacted with active methylene (compound 3) to form monomer 1 (M1) with a knoevenagel reaction. Finally, the corresponding polymers were obtained by Stille reaction between M1 and different copolymerization units including monomer 2 (M2), monomer 3 (M3), or monomer 4 (M4), respectively. We provided ^1^H nuclear magnetic resonance spectroscopy for those compounds and semiconducting polymers as shown in Figs. S2 to S6. Furthermore, the density functional theory calculation of NIR-1 showed that the highest occupied molecular orbital (HOMO) was delocalized on the fused molecule core, and the lowest unoccupied molecular orbital (LUMO) was delocalized across the backbone. Due to the multiple ICT, the separation of HOMO-LUMO occurred, extending absorption wavelength (Fig. 2C).

We investigated the optical properties of those semiconducting polymers in the organic solvent. As shown in Fig. 2D, the maximum absorption peaks of those polymers in toluene were around 780 to 800 nm, during which NIR-1 showed the highest absorption peak. Those polymers showed strong fluorescence emission from 900 to 1,100 nm, where NIR-1 displayed the strongest fluorescence intensity (Fig. 2E). The QYs of these polymers in toluene were determined to be 3.35%, 2.17%, or 3.32% for NIR-1, NIR-2, or NIR-3, respectively, using IR26 in 1,2-dichloroethane (QY = 0.05%) as reference (Table S3).

### Construction of NIR-II fluorescent semiconducting polymer nanoparticles

To improve the biological safety and circulation time in blood, we converted the semiconducting polymers into semiconducting polymer nanoparticles (SPNs) via DSPE-PEG_2000_, using the nanoprecipitation method (Fig. 2B and Fig. S7). Typically, SPNs(NIR-1) was found to be roughly spherical shape as confirmed by a transmission electron microscope (TEM) (Fig. 2H), with dynamic light scattering size of 43 to 50 nm (Fig. S8) and exhibited an excellent colloidal stability within 21 d in water, 1 × phosphate-buffered saline buffer, 0.9% saline solution, and cell culture medium containing 10% fetal bovine serum (Fig. S9). The absorption peaks of SPNs(NIR-1), SPNs(NIR-2), and SPNs(NIR-3) (10 μg/ml) were 780, 830, and 800 nm, respectively (Fig. 2F). Interestingly, these SPNs exhibited color variation from gray green, pale green, to pale blue (Fig. 2F, inset). Moreover, SPNs(NIR-1), SPNs(NIR-2), and SPNs(NIR-3) (10 μg/ml) showed strong fluorescent emission from 900 to 1,100 nm, where SPNs(NIR-1) presented the highest fluorescent peak (Fig. 2G).

To investigate NIR-II fluorescent performance of those SPNs, ICG was used as a reference (Fig. 2I to L). SPNs(NIR-1) in water displayed the strongest fluorescence intensity, which is 2.55-fold, 2.94-fold, or 5.72-fold that of SPNs(NIR-2), SPNs(NIR-3), or ICG, with the same concentration (50 μg/ml), respectively, under excitation of 808 nm (Fig. 2I and J). Moreover, the fluorescent intensity of SPNs(NIR-1) increased as the concentration of SPNs(NIR-1) increased from 10 to 100 μg/ml (Fig. S10 and Fig. 2K). Furthermore, those SPNs exhibited slight attenuation of fluorescence after continuous irradiation of 808-nm laser, while the fluorescence of ICG was greatly attenuated (Fig. S11 and Fig. 2L). Considering the strongest fluorescence signal and robust fluorescence stability, SPNs(NIR-1) was chosen for the later experiment.

### Construction of NIR-II /MRI hybrid nanoparticles

Firstly, SPIO nanoparticles were synthesized using the pyrolysis method in the organic solvent [26]. The representative TEM image displayed monodispersion nanoparticles with average size of about 3 nm in the dry state (Fig. S12). To integrate multimodal NIR-II fluorescence/MRI, hybrid semiconducting polymer nanoparticles @ superparamagnetic iron oxide (SPNs(NIR-1)@SPIO) were prepared via the 1-step nanoprecipitation method (Fig. 2B). TEM images showed that ultrasmall iron oxide nanoparticles were embedded into SPNs (Fig. 2M). Dynamic light scattering size displayed the average hydrodynamic size was about 58 nm (Fig. S13).

Then, the longitudinal (*r_1_*) and transversal (*r_2_*) relaxivities of SPNs(NIR-1)@SPIO were measured in solution via a Bruker Minispec MQ60 nuclear magnetic resonance analyzer. *r_2_* was measured to be 17.544 mM^−1^s^−1^, while *r_1_* was calculated to be 0.452 mM^−1^s^−1^, yielding an *r_2_*/*r_1_* ratio of 38.8 (Fig. 2N). Next, the aqueous solution of SPNs(NIR-1)@SPIO was scanned by 7-T MRI scanner. From both T_2_-weighted MR images, the images became gradual darkness, indicating negative contrast (Fig. 2O). As concentration increased, both T_1_- and T_2_-weighted MRI signal intensities of SPNs(NIR-1)@SPIO gradually decreased (Fig. S14). Besides, NIR-II images of SPNs(NIR-1)@SPIO showed that the NIR-II fluorescent signal increased with increasing concentration (Fig. 2P and Fig. S15). Those results suggested the potential of SPNs(NIR-1)@SPIO for dual-modality MR and NIR-II fluorescence imaging.

### NIR-II fluorescence imaging and MRI of carotid atherosclerosis plaque

**Fig. 3.**
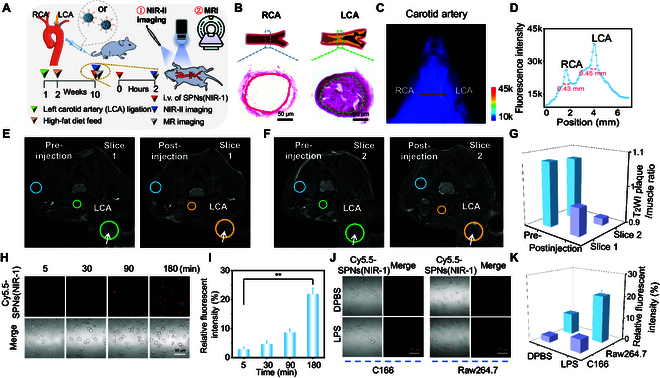
NIR-II fluorescence imaging and MRI of carotid atherosclerosis plaque. (A) Schematic diagram for preparation of mice mode with atherosclerotic plaque of carotid artery (AS mice) and NIR-II fluorescence/MRI imaging. (B) The H&E-stained images of RCA and LCA. Green curve indicated plaque in lumen. (C) NIR-II fluorescence imaging of cervical region of AS mice post intravenously injected with SPNs(NIR-1). (D) The fluorescence intensity profiles along the red dashed line in RCA and LCA (C). (E and F) Representative T_2_WI images of cervical region of 2 slices of AS mice pre- and postinjection (24 h) of SPNs(NIR-1)@SPIO. Inset pictures enlarged the LCA (green or orange circles), white arrows indicated plaque, and blue circles indicated muscle. (G) The T_2_WI plaque-to-muscle ratio in (E) and (F). (H) Representative confocal images of Raw264.7 cells incubated with Cy5.5-SPNs(NIR-1) for various times. (I) The relative fluorescence intensity of Cy5.5-SPNs(NIR-1) in (H). (J) Representative confocal images of C166 and RAW264.7 cells pretreated with LPS or not and incubated with Cy5.5-SPNs(NIR-1), respectively. (K) The relative fluorescence intensity (%) of Cy5.5-SPNs(NIR-1) in (J).

To prepare atherosclerotic plaque in left carotid artery (LCA), the LCA of C57BL/6 adult male mice was ligated, followed by 8 weeks of high-fat feed (Fig. 3A). The hematoxylin-eosin (H&E) staining images verified that the atherosclerotic plaques were formed locally in LCA, while the undisturbed right carotid artery (RCA) remained patency and plaque-free (Fig. 3B). To identify the localization and detail in plaque lesion, the C57BL/6 mice were administered with SPNs(NIR-1) or SPNs(NIR-1)@SPIO via intravenous injection, and then NIR-II fluorescence imaging and MRI of carotid atherosclerosis mice, respectively, was performed.

From whole-body NIR-II fluorescence images, we observed clear vasculature throughout whole body, large and medium blood vessels in the torso and limbs of mice, indicating the good resolution for SPNs(NIR-1) as NIR-II probes (Fig. S16). We detected carotid arteries in the supine position and lateral saphenous vein in prone position (Fig. S17). These results demonstrated good spatial resolution of SPNs(NIR-1) for NIR-II fluorescence imaging of vascular system in vivo. The atherosclerosis mice bearing plaque in LCA was also intravenously injected with SPNs(NIR-1). Importantly, we noted the fluorescent intensity of LCA was higher than that of RCA, confirming SPNs(NIR-1) was accumulated into plaque in LCA after SPNs(NIR-1) intravenous injection (Fig. 3C and D and Fig. S18).

To explore MRI imaging of carotid atherosclerosis plaque, the atherosclerosis mice bearing plaque was intravenously injected with SPNs(NIR-1)@SPIO. Here, we collected consecutive T_2_-weighted images (T_2_WI) of cervical region in the transverse direction to recognize detail of carotid arteries, and 2 random nonconsecutive sections were analyzed (Fig. 3E and F). The enrichment of SPNs(NIR-1)@SPIO in LCA might help recognize plaque lesion via comparing pre- (green circles) and postinjection (orange circles) images. From 2 sections T_2_WI image at 24 h post injection, the lipid pool in plaque (white arrows) became slightly darkness (orange circle), in contrast to that of preinjection (green circles) (Fig. 3E and F, inset). The results of previous studies suggested that quantitative evaluation using the plaque/muscle ratio index panel had a probability to predict the risk of carotid plaque.^[^^27^^]^ By comparing pre- and postinjection (24 h) T_2_WI plaque images, we attempted to calculate the plaque/muscle ratio for carotid plaques. Here, we found that T_2_WI plaque/muscle ratio of AS mice decreased from 1.078 to 0.972 in slice 1 and 1.071 to 0.918 in slice 2, at 24 h postinjection of SPNs(NIR-1)@SPIO, respectively (Fig. 3G). We then located SPIO in the plaque via Prussian blue iron stain. The stain demonstrated the blue granules of ferro debris particles in plaque (Fig. S19). This result supported the enrichment of SPNs(NIR-1)@SPIO in plaque. In summary, plaque/muscle ratio on T_2_WI in MRI imaging using SPNs(NIR-1)@SPIO might be a useful predictor of carotid plaques.

Considering macrophage as target for indicating vulnerable plaques, we explored the cellular uptake of macrophage toward SPNs(NIR-1). To trace the probe distribution in macrophages (Raw264.7) via fluorescent confocal imaging, we employed cyanine dye Cy5.5 (λ_ex_ = 675 nm, λ_em_ = 694 nm) to label NIR-1 via inserting DSPE-PEG_2000_-Cy5.5 into SPNs (DSPE-PEG_2000_-Cy5.5:DSPE-PEG_2000_ = 1:10), and obtained Cy5.5-SPNs(NIR-1) [28]. From confocal images, we observed a time-dependent phagocytosis of Cy5.5-SPNs(NIR-1). The fluorescent intensity from cells gradually enhanced, as the incubation time extending from 5 to 180 min (Fig. 3H and I), indicating that macrophages could rapidly endocytose Cy5.5-SPNs(NIR-1).

Furthermore, we studied the endocytosis difference between vascular epithelial cells and phagocytes in vitro, via incubating Raw264.7 and vascular endothelial cells (C166) with Cy5.5-SPNs(NIR-1). Since proinflammatory mediators have multiple negative effects on the development of atherosclerotic plaque [2,24], we examined whether inflammatory stimulation affected phagocytic capability of macrophages to Cy5.5-SPNs(NIR-1). From confocal images, when the macrophages were pretreated with lipopolysaccharide (LPS) (0.1 μg/ml), we observed that the internalization of Cy5.5-SPNs(NIR-1) in RAW264.7 cells remarkably increased after 90-min incubation. By contrast, there was no obviously increase in cellular uptake of Cy5.5-SPNs(NIR-1) for C166 cells, even after LPS treatment (Fig. 3J and K). These results demonstrated that Cy5.5-SPNs(NIR-1) could be effectively endocytosed by macrophage rather than epithelial cells.

### Distinguishing unstable plaque in vivo via NIR-II imaging

**Fig. 4.**
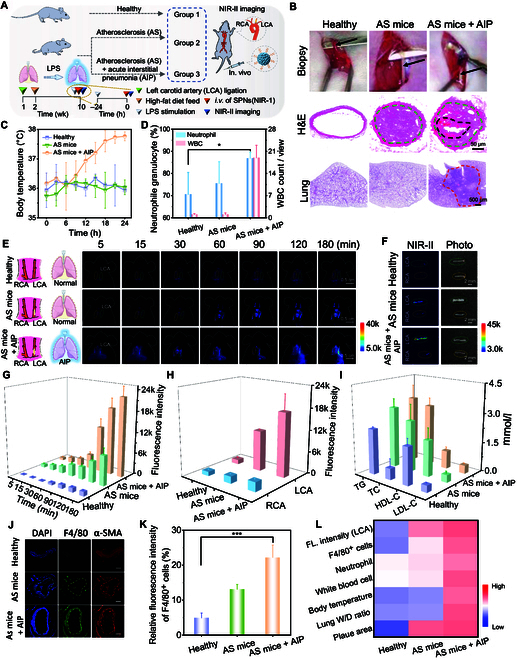
Distinguishing unstable plaque in vivo via NIR-II imaging. (A) Schematic diagram for NIR-II fluorescence imaging of carotid plaque in AS mice and AS mice + AIP, using healthy mice as control, after intravenous injection of SPNs(NIR-1) (*n* = 3). (B) Representative biopsy of LCAs seperated from healthy mice, AS mice, and AS mice + AIP, as well as corresponding H&E-stained images of LCAs and lungs. Green curve indicated plaque, black curve indicated a soft lipid pool, and red curve indicated lung interstitial inflammation. (C) The body temperature changes in various groups. (D) The neutrophile granulocyte (%) and WBC count for various groups. (E and F) NIR-II fluorescence images of healthy mice, AS mice, and AS mice + AIP at different times post intravenous injection of SPNs(NIR-1), and NIR-II fluorescence images of ex vivo carotid arteries for various groups. (G) The fluorescence intensity of LCAs at different time points for various groups in (E). (H) The fluorescence intensity of LCAs and RCAs for various groups in (F). (I) The TG, TC, HDL-C, and low-density lipoprotein control (LDL-C) levels for various groups. (J) Representative fluorescence confocal images of ex vivo carotid arteries, stained by DAPI (nucleus), F4/80 (macrophage), and α-SMA (smooth muscle actin). Blue, green, and red curves indicated lumens. (K) The relative fluorescence intensity of F4/80^+^ cells in (J). (L) Heat map for fluorescence intensity of LCA, F4/80^+^ cells, neutrophile, WBC, body temperature, lung wet/dry (W/D) ratio, and plaque area.

Clinical studies have shown that inflammation can exacerbate plaque progression and instability, among which, pneumonia is an important trigger for atherosclerosis-related cardiovascular diseases or comorbidities [29]. Here, we performed NIR-II fluorescence imaging of carotid plaque in atherosclerosis mice (named AS mice) and atherosclerosis mice combined with acute interstitial pneumonia (named AS mice + AIP), respectively, using healthy mice as control (Fig. 4A). We analyzed the pathological features of carotid artery and lung tissue from healthy mice, AS mice, and AS mice + AIP. The H&E-stained images of left carotid arteries confirmed plaque-free vessel for healthy mice, atherosclerotic vascular narrowing and plaque (green dot line) for AS mice, and a large soft lipid pool (black dot line) in plaque (green dot line) for AS mice + AIP, respectively (Fig. 4B). The plaque areas (%, mean) was calculated to be 81% and 84% for AS mice and AS mice + AIP, respectively (Fig. S20).

Moreover, the representative lung H&E staining images confirmed the existing interstitial edema and inflammation in AS mice + AIP, while no obvious inflammation in healthy or AS mice (Fig. 4B). Besides, compared with AS mice or healthy mice, AS mice + AIP exhibited the increased body temperature (Fig. 4C), neutrophils, and white blood cells (WBCs) of peripheral blood (Fig. 4D and Fig. S21) and wet-to-dry lung ratio (Fig. S22), as well as increased erythrocyte sedimentation rate, C-reactive protein, and procalcitonin (Fig. S23), which all supported the pulmonary inflammation in LPS-treated mice.

To test the ability of SPNs(NIR-1) for NIR-II fluorescence imaging of unstable plaques in carotid artery, healthy mice, AS mice, and AS+AIP mice were intravenously injected with SPNs(NIR-1), followed by NIR-II fluorescence imaging of cervical region. From NIR-II fluorescent images, we found that the fluorescence signals from LCA for AS mice and AS + AIP mice gradually enhanced from 5 to 180 min (Fig. 4E). Moreover, the fluorescence signals of LCA for AS mice +AIP were noticeably higher than those of AS mice and healthy mice since 90 min. In detail, the fluorescence signals of LCA for AS mice + AIP mice were 3-fold or 15-fold that of AS mice or healthy mice at 120 min postinjection, respectively, from quantification results (Fig. 4G). In contrast, the fluorescence signals of right carotid arteries of 3 groups were not remarkably elevated (Fig. S24). Then, we performed regional anatomy of the neck and observed and imaged in living mouse models (healthy mice and AS mice). The larger gland completely masked the fluorescence signals of the circulated nanoprobes in carotid veins, while the less-covered muscle on the carotid arteries did not affect fluorescence imaging (Fig. S25). Thus, these results supported that the neck fluorescence signals mainly came from the carotid arteries.

Subsequently, we imaged the excised carotid arteries from sacrificed mice at 6 h post intravenous injection. The fluorescence signals of LCA were dramatically higher than that of RCA in both AS mice and AS mice + AIP, respectively, indicating the strong accumulation of NIR-II nanoprobes in LCA (Fig. 4F). Moreover, we found LCA fluorescence signal for AS mice +AIP was higher than that of AS mice and healthy mice (Fig. 4H). These results confirmed the consistency of NIR-II fluorescence imaging plaque between in vivo and ex vivo.

Elevated cholesterol levels and triglyceride (TG) had been demonstrated to increase arterial inflammation and atherosclerotic plaque burden, while high-density lipoprotein-cholesterol (HDL-C) had anti-inflammatory effects, thus protecting endo-thelial cell functions [1,30]. Here, we studied the serum lipid level and inflammatory indicators for those mice. AS mice and AS mice + AIP exhibited the increased level of TG and total cholesterol (TC), in contrast to healthy mice (Fig. 4I). Notably, AS mice + AIP showed a less HDL-C level than that of AS mice or healthy mice, indicating decreased cholesterol removing abilities, which might lead to atherosclerotic lesion burden [31].

Furthermore, we examined the infiltration of macrophage in carotid arteries via immunofluorescence staining of nucleic acid dye 4′,6-diamidino-2-phenylindole (DAPI), F4/80 (macrophage), and α-SMA (smooth muscle actin). From confocal images of left carotid arteries and their quantification (Fig. 4J and K), we found more macrophages infiltrated in AS mice and AS mice + AIP, compared with that of healthy mice. Thus, we drew the heat map for healthy mice, AS mice, and AS +AIP mice, including those parameters such as fluorescence intensity of left carotid arteries and right carotid arteries, F4/80 (macrophage) proportion, neutrophile, WBCs, body temperature (B.T), lung wet/dry (W/D) ratio, and plaque area proportion (Fig. 4L). From the heat map, the NIR-II fluorescence signals of left carotid arteries in healthy mice, AS mice and AS mice + AIP showed good correlation with macrophage infiltration, inflammatory indicator and plaque area.

### Imaging IPH in vivo

**Fig. 5.**
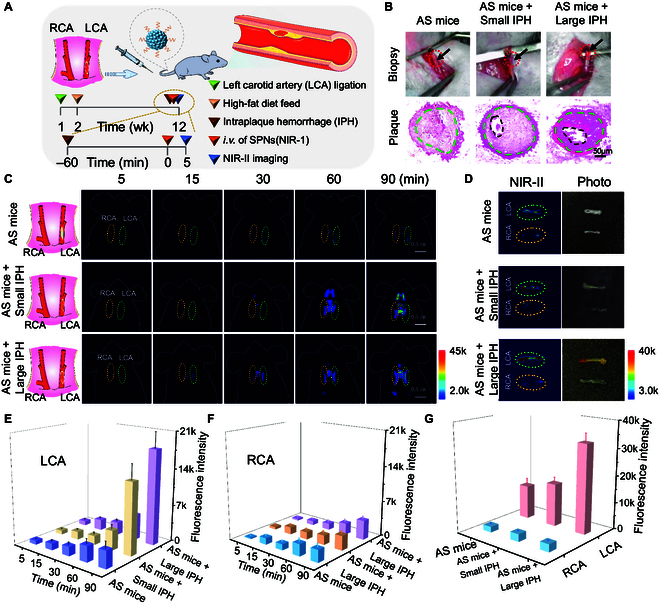
Imaging IPH in vivo. (A) Schematic diagram for NIR-II fluorescence imaging of atherosclerosis mice (named AS mice) and atherosclerosis mice combined with small IPH (named AS + small IPH) and large IPH (named AS + large IPH), after intravenous injection of SPNs(NIR-1) (*n* = 3). (B) Representative biopsy of LCA from AS mice, AS mice + small IPH, and AS mice + large IPH, as well as corresponding H&E-stained images of LCAs. The marked area by green curve indicated plaque, and black curve indicated blood micro-injection. (C and D) NIR-II fluorescence images of AS mice, AS mice + small IPH, and AS mice + large IPH at different times postinjection of SPNs(NIR-1), and NIR-II fluorescence images of ex vivo carotid arteries. Green curve indicated LCAs; orange curve indicated RCAs. (E and F) The fluorescence intensity of LCAs and RCAs at different time points for various groups in (C). (G) The fluorescence intensity of LCAs and RCAs for various groups in (D).

IPH can accelerate the destabilization of plaque via free cholesterol secretion, macrophage accumulation and necrotic core expansion, which is regarded as a feature of advanced and rapidly progressing plaques [22,32]. Herein, we tested the ability of SPN(NIR-1) for identifying IPH in living mice via NIR-II fluorescence imaging. To prepare an IPH model, atherosclerotic plaques in LCA was micro-injected with small or large dose of fresh blood; meanwhile, AS mice were used as control (Fig. 5A) [33]. Correspondingly, surgical exploration revealed obvious intraplaque bleeding spots in small IPH or large IPH mice. Representative H&E staining images of left carotid arteries confirmed the presence of IPH within plaques after local injection of fresh blood (Fig. 5B). For NIR-II fluorescence imaging, mice were intravenously injected with SPN(NIR-1) at 1 h post IPH. From fluorescence images (Fig. 5C), the fluorescence signal of LCA for IPH mice presented a sharp increase at 90 min, which was higher than that of AS mice. Moreover, the fluorescence of left carotid arteries for large IPH mice was higher signal than that of small IPH at 90 min (Fig. 5E). Conversely, the right carotid arteries, i.e. without IPH, showed much smaller increase of fluorescence signal, compared with that of LCAs (Fig. 5F).

Furthermore, we excised the carotid arteries for NIR-II fluorescence imaging at 6 h post intravenous injection. From the representative ex vivo images, the fluorescence intensity of LCA for AS mice + large IPH was 1.7-fold and 4.17-fold that of AS mice + small IPH and AS mice, respectively (Fig. 5D and G). In contrast, the fluorescent signals of RCA with plaque-free arteries were negligible for those 3 groups. Thus, these results demonstrated the consistency between fluorescence signals in carotid arteries and the degree of IPH, which confirmed the great potential of SPN(NIR-1) for NIR-II imaging of IPH in mice and was beneficial for early imaging vulnerable plaque and giving an early-warning sign.

In addition, we tested the cytotoxicity of SPNs(NIR-1) via 3-(4,5-dimethylthiozol-2-yl)-2,5-diphenyl tetrazolium bromide (MTT) assays and found SPNs(NIR-1) induced no effect on the cellular viability, suggesting good biocompatibility (Fig. S26). After NIR-II fluorescent imaging, we collected the main organs. H&E staining of main organs demonstrated no obvious biological toxicity induced by SPNs(NIR-1) after multiple imaging (Fig. S27).

## Discussion

During the disease of hypertension, diabetes, hyperlipidemia, infection, etc., the risk of plaque rupture or thrombus formation would be increased, where the macrophages act as key roles in atherogenesis, formation of foam cell, and generation of proteinase that lead to plaque destabilization and rupture [4,34]. Specifically, macrophages engulfed lipoproteins in intima to form lipid-laden foam cells, leading to early atherosclerotic lesions [2,24]. If the pro-inflammatory microenvironment persisted, such as reactive oxygen species, changes in pH and protein phosphorylation state, the atherosclerotic lesion would progress to an advanced stage characterized by increased macrophage apoptosis and defective clearance of apoptotic cells [14,35]. Besides, macrophages, mast cells, T cells and foam cells could induce inflammation and therefore create a large lipid core within plaque, leading to IPH, which is more prevalent at the symptomatic side in patients with carotid plaques and <50% stenosis [1,2]. This catastrophic combination promoted the plaque necrosis, which was a key feature of vulnerable plaque. The plaque necrosis further triggered occlusive luminal thrombosis and severe emergencies, namely, myocardial infarction, stroke, and sudden cardiac death [5,36]. Thus, macrophage-associated pathological process an important target for both diagnosis and therapy for atherosclerosis.

Fluorescence imaging in the state-of-the-art NIR-II region with good penetration depth and high resolution is promising for imaging carotid artery [37]. This work illustrated the feasibility of developing a biocompatible and stable NIR-II fluorescence nanoprobe for high-resolution imaging of vessels and vascular-related lesions such as vulnerable plaque and IPH in carotid artery. To achieve this, semiconducting polymer-based nanoprobes with NIR-II fluorescent emission was synthesized. In real-time imaging of living mice, SPNs(NIR-1) could distinguish stable plaque from inflammatory-induced unstable plaques through different fluorescence insensities.

Importantly, the real-time monitoring of IPH in carotid artery was achieved via NIR-II fluorescence imaging by SPN(NIR-1), which was rarely tried using fluorescence imaging in previous reports. Taken together, SPN(NIR-1) could clearly outline the pathological characteristics of high-risk atherosclerotic plaques in carotid artery. Although the fluorescence imaging demonstrated here was based on passive targeting, these results still confirmed the great potential of SPN(NIR-1) for NIR-II imaging vulnerable plaques.

Compared with traditional imaging technologies, nanoprobes can specifically accumulate at atherosclerotic plaques through cytophagy or modification of surface moieties. Considering that the carotid artery is easy to be monitored by imaging technics due to its superficial anatomical location, nanoprobes in the NIR-II region exhibit a high translational potential for imaging carotid plaque, while MRI imaging exhibits clinical values for plaque details. The combination of purposive accumulation coupled with multimodal imaging techniques is going to further improve both the sensitivity and accuracy of imaging diagnosis in atherosclerotic plaques. Beyond high-resolution imaging and real-imaging potential, nanoprobes could also be taken as an active part in the therapy of angiocardiopathy (such as high-risk plaques and thrombosis) by different strategies, including anti-inflammation, antioxidation, and repair of damaged vascular intima.

In summary, we screened a series of NIR-II fluorescent polymers with strong absorption/emission, selected a preferential polymer with good NIR-II fluorescent QY, and prepared them into nanoprobes. Importantly, SPN(NIR-1) revealed the superior advantages in spatial resolution for NIR-II fluorescence imaging of angiography in carotid artery. Moreover, we employed SPN(NIR-1) for distinguishing vulnerable plaque from stable plaque and IPH via the dynamic change of fluorescence signal in carotid artery. Notably, the local change of fluorescence intensity within arteries were well correlated with the pathological characteristics of plaque. Furthermore, we prepared hybrid NIR-II/MRI multimodel probe by incorporating ultrasmall SPIO into NIR-II nanoprobe and provided an effective tool for spatial imaging of carotid plaque. Thus, such novel vascular imaging platform can be used to monitor pathological change in plaques at an early stage, providing a powerful imaging tool for monitoring and early warning high-risk atherosclerotic plaques in carotid artery.

## Methods

The methods can be found in the Supplementary Materials.

## Supporting Information

Imaging Carotid Plaque Burden in living mice via Hybrid Semiconducting Polymer Nanoparticles Based Near-Infrared-II Fluorescence and Magnetic Resonance Imaging.

A NIR-II dye named NIR-1 was designed and synthesized probe 1, i.e., SPNs(NIR-1), and hybrid NIR-II/MRI multimodel nanoprobe probe 2, i.e., SPNs(NIR-1)@SPIO, was synthesized via incorporating ultrasmall SPIO into NIR-II nanoprobe. Two probes were applied for NIR-II imaging or magnetic resonance imaging of carotid atherosclerotic plaque in vivo.

## Data Availability

The datasets used and/or analyzed during the current study are available from the author on reasonable request.

## References

[1] Libby P. The changing landscape of atherosclerosis. Nature. 2021;592(7855):524. 33883728 10.1038/s41586-021-03392-8

[2] Engelen SE, Robinson AJB, Zurke YX, Monaco C. Therapeutic strategies targeting inflammation and immunity in atherosclerosis: How to proceed? Nat Rev Cardiol. 2022;19(8):522. 35102320 10.1038/s41569-021-00668-4 PMC8802279

[3] Donnan GA, Fisher M, Macleod M, Davis SM. Stroke. Lancet. 2008;371(9624):1612–1623. 18468545 10.1016/S0140-6736(08)60694-7

[4] Sakakura K, Nakano M, Otsuka F, Ladich E, Kolodgie FD, Virmani R. Pathophysiology of atherosclerosis plaque progression. Heart Lung Circ. 2013;22(6):399–411. 23541627 10.1016/j.hlc.2013.03.001

[5] Arbab-Zadeh A, Fuster V. From detecting the vulnerable plaque to managing the vulnerable patient: JACC state-of-the-art review. J Am Coll Cardiol. 2019;74(12):1582–1593. 31537269 10.1016/j.jacc.2019.07.062

[6] Bonati LH, Jansen O, de Borst GJ, Brown MM. Management of atherosclerotic extracranial carotid artery stenosis. Lancet Neurol. 2022;21(3):273–283. 35182512 10.1016/S1474-4422(21)00359-8

[7] Canfran-Duque A, Rotllan N, Zhang X, Andres-Blasco I, Thompson BM, Sun J, Price NL, Fernandez-Fuertes M, Fowler JW, Gomez-Coronado D, et al. Macrophage-derived 25-hydroxycholesterol promotes vascular inflammation, atherogenesis, and lesion remodeling. Circulation. 2023;147(5):388–408. 36416142 10.1161/CIRCULATIONAHA.122.059062 PMC9892282

[8] Boada C, Zinger A, Tsao C, Zhao P, Martinez JO, Hartman K, Naoi T, Sukhoveshin R, Sushnitha M, Molinaro R, et al. Rapamycin-loaded biomimetic nanoparticles reverse vascular inflammation. Circ Res. 2020;126(1):25–37. 31647755 10.1161/CIRCRESAHA.119.315185

[9] Yao J, Yang Z, Huang L, Yang C, Wang J, Cao Y, Hao L, Zhang L, Zhang J, Li P, et al. Low-intensity focused ultrasound-responsive ferrite-encapsulated nanoparticles for atherosclerotic plaque neovascularization theranostics. Adv Sci (Weinh). 2021;8(19): Article e2100850. 34382370 10.1002/advs.202100850 PMC8498883

[10] Khraishah H, Jaffer FA. Intravascular molecular imaging to detect high-risk vulnerable plaques: Current knowledge and future perspectives. Curr Cardiovasc Imaging Rep. 2020;13:8.

[11] Sardar P, Chatterjee S, Aronow Herbert D, Kundu A, Ramchand P, Mukherjee D, Nairooz R, Gray William A, White Christopher J, Jaff Michael R, et al. Carotid artery stenting versus endarterectomy for stroke prevention: A meta-analysis of clinical trials. J Am Coll Cardiol. 2017;69(18):2266–2275. 28473130 10.1016/j.jacc.2017.02.053

[12] Qiao Y, Etesami M, Malhotra S, Astor BC, Virmani R, Kolodgie FD, Trout HH 3rd, Wasserman BA. Identification of intraplaque hemorrhage on MR angiography images: A comparison of contrast-enhanced mask and time-of-flight techniques. AJNR Am J Neuroradiol. 2011;32(3):454–459. 21233234 10.3174/ajnr.A2320 PMC3337083

[13] Bos D, van Dam-Nolen, DHK, Gupta A, Saba L, Saloner D, Wasserman BA, van der Lugt, A. Advances in multimodality carotid plaque imaging: AJR expert panel narrative review. AJR Am J Roentgenol. 2021;217:16. 33438455 10.2214/AJR.20.24869

[14] Chan J, Dodani SC, Chang CJ. Reaction-based small-molecule fluorescent probes for chemoselective bioimaging. Nat Chem. 2012;4(12):973–984. 23174976 10.1038/nchem.1500 PMC4096518

[15] Ma Y, Ma Y, Gao M, Han Z, Jiang W, Gu Y, Liu Y. Platelet-mimicking therapeutic system for noninvasive mitigation of the progression of atherosclerotic plaques. Adv Sci. 2021;8(8): Article 2004128. 10.1002/advs.202004128 PMC8061396 33898191

[16] Verjans JW, Osborn EA, Ughi GJ, Calfon Press MA, Hamidi E, Antoniadis AP, Papafaklis MI, Conrad MF, Libby P, Stone PH, et al. Targeted near-infrared fluorescence imaging of atherosclerosis: Clinical and intracoronary evaluation of indocyanine green. JACC Cardiovasc Imaging. 2016;9(9):1087–1095. 27544892 10.1016/j.jcmg.2016.01.034 PMC5136528

[17] Ye Z, Ji M, Wu K, Yang J, Liu AA, Sun W, Ding D, Liu D. In-sequence high-specificity dual-reporter unlocking of fluorescent probe enables the precise identification of atherosclerotic plaques. Angew Chem Int Ed. 2022;61(29): e202204518. 10.1002/anie.202204518 35460326

[18] Situ B, Gao M, He X, Li S, He B, Guo F, Kang C, Liu S, Yang L, Jiang M, et al. A two-photon AIEgen for simultaneous dual-color imaging of atherosclerotic plaques. Mater Horiz. 2019;6:546.

[19] Albaghdadi MS, Ikegami R, Kassab MB, Gardecki JA, Kunio M, Chowdhury MM, Khamis R, Libby P, Tearney GJ, Jaffer FA. Near-infrared autofluorescence in atherosclerosis associates with ceroid and is generated by oxidized lipid-induced oxidative stress. Arterioscler Thromb Vasc Biol. 2021;41(7):e385–e398. 34011166 10.1161/ATVBAHA.120.315612 PMC8222195

[20] Xie Z, Yang Y, He Y, Shu C, Chen D, Zhang J, Chen J, Liu C, Sheng Z, Liu H, et al. *In vivo* assessment of inflammation in carotid atherosclerosis by noninvasive photoacoustic imaging. Theranostics. 2020;10(10):4694–4704. 32292523 10.7150/thno.41211 PMC7150488

[21] Wang Z, Wang X, Wan J-B, Xu F, Zhao N, Chen M. Optical imaging in the second near infrared window for vascular bioimaging. Small. 2021;17(43): Article e2103780. 34643028 10.1002/smll.202103780

[22] Zhao X-Q, Sun J, Hippe DS, Isquith DA, Canton G, Yamada K, Balu N, Crouse JR 3rd, Anderson TJ, Huston J 3rd, et al. Magnetic resonance imaging of intraplaque hemorrhage and plaque lipid content with continued lipid-lowering therapy: Results of a magnetic resonance imaging substudy in AIM-HIGH. Circ Cardiovasc Imaging. 2022;15(11): Article e014229. 36378778 10.1161/CIRCIMAGING.122.014229 PMC9773914

[23] Carbone F, Teixeira PC, Braunersreuther V, Mach F, Vuilleumier N, Montecucco F. Pathophysiology and treatments of oxidative injury in ischemic stroke: Focus on the phagocytic NADPH oxidase 2. Antioxid Redox Signal. 2015;23(5):460–489. 24635113 10.1089/ars.2013.5778 PMC4545676

[24] Song JW, Nam HS, Ahn JW, Park HS, Kang DO, Kim HJ, Kim YH, Han J, Choi JY, Lee SY, et al. Macrophage targeted theranostic strategy for accurate detection and rapid stabilization of the inflamed high-risk plaque. Theranostics. 2021;11(18):8874–8893. 34522216 10.7150/thno.59759 PMC8419038

[25] Qiao H, Wang Y, Zhang R, Gao Q, Liang X, Gao L, Jiang Z, Qiao R, Han D, Zhang Y, et al. MRI/optical dual-modality imaging of vulnerable atherosclerotic plaque with an osteopontin-targeted probe based on Fe3O4 nanoparticles. Biomaterials. 2017;112:336–345. 27788352 10.1016/j.biomaterials.2016.10.011

[26] Yue RY, Zhang C, Xu L, Wang YJ, Guan GQ, Lei LL, Zhang XB, Song GS. Dual key co-activated nanoplatform for switchable MRI monitoring accurate ferroptosis-based synergistic therapy. Chem. 2022;8:1956.

[27] Matsumoto K, Ehara S, Hasegawa T, Sakaguchi M, Otsuka K, Yoshikawa J, Shimada K. Localization of coronary high-intensity signals on t1-weighted mr imaging: relation to plaque morphology and clinical severity of angina pectoris. J Am Coll Cardiol. 2015;8(10):1143–1152. 10.1016/j.jcmg.2015.06.013 26363839

[28] Yang X, Li X, Liu L, Chen YH, You Y, Gao Y, Liu YY, Yang L, Tong K, Chen DS, et al. Transferrin-Pep63-liposomes accelerate the clearance of Aβ and rescue impaired synaptic plasticity in early Alzheimer's disease models. Cell Death Discov. 2021;7(1): Article 256. 34548476 10.1038/s41420-021-00639-1 PMC8455582

[29] Grzegorowska O, Lorkowski J. Possible correlations between atherosclerosis, acute coronary syndromes and COVID-19. J Clin Med. 2020;9(11): Article 3746. 33233333 10.3390/jcm9113746 PMC7700642

[30] Navab M, Reddy ST, Van Lenten BJ, Fogelman AM. HDL and cardiovascular disease: Atherogenic and atheroprotective mechanisms. Nat Rev Cardiol. 2011;8(4):222–232. 21304474 10.1038/nrcardio.2010.222

[31] Sun J, Zhao X-Q, Balu N, Neradilek B, Isquith DA, Yamada K, Canton G, Crouse JR III, Anderson TJ, Huston J III, et al. Carotid plaque lipid content and fibrous cap status predict systemic CV outcomes: The MRI substudy in AIM-HIGH. JACC Cardiovasc Imaging. 2017;10(3):241–249. 28279371 10.1016/j.jcmg.2016.06.017 PMC5347460

[32] Crombag G, Aizaz M, Schreuder F, Benali F, van Dam-Nolen DHK, Liem MI, Lucci C, van der Steen AF, Daemen M, Mess WH, et al. Proximal region of carotid atherosclerotic plaque shows more intraplaque hemorrhage: The plaque at risk study. AJNR Am J Neuroradiol. 2022;43(2):265–271. 35121587 10.3174/ajnr.A7384 PMC8985675

[33] Daeichin V, Sluimer JC, van der Heiden K, Skachkov I, Kooiman K, Janssen A, Janssen B, Bosch JG, de Jong N, Daemen MJAP, et al. Live observation of atherosclerotic plaque disruption in apolipoprotein E-deficient mouse. Ultrasound Int open. 2015;1(2):E67–E71. 27689156 10.1055/s-0035-1565092 PMC5023208

[34] Narula N, Olin JW, Narula N. Pathologic disparities between peripheral artery disease and coronary artery disease. Arterioscler Thromb Vasc Biol. 2020;40(9):1982–1989. 32673526 10.1161/ATVBAHA.119.312864

[35] Li J, Zhao N, Zhang W, Li P, Yin X, Zhang W, Wang H, Tang B. Assessing the progression of early atherosclerosis mice using a fluorescence nanosensor for the simultaneous detection and imaging of pH and phosphorylation. Angew Chem Int Ed. 2022;62(3): Article e202215178. 10.1002/anie.202215178 36357335

[36] Li X, Li J, Wu G. Relationship of neutrophil-to-lymphocyte ratio with carotid plaque vulnerability and occurrence of vulnerable carotid plaque in patients with acute ischemic stroke. Biomed Res Int. 2021;2021: Article 6894623. 34250090 10.1155/2021/6894623 PMC8238559

[37] Abran M, Stahli BE, Merlet N, Mihalache-Avram T, Mecteau M, Rheaume E, Busseuil D, Tardif JC, Lesage F. Validating a bimodal intravascular ultrasound (IVUS) and near-infrared fluorescence (NIRF) catheter for atherosclerotic plaque detection in rabbits. Biomed Opt Express. 2015;6(10):3989–3999. 26504648 10.1364/BOE.6.003989 PMC4605057

