# Supplementary material for: Imaging Carotid Plaque Burden in Living Mice via Hybrid Semiconducting Polymer Nanoparticles-Based Near-Infrared-II Fluorescence and Magnetic Resonance Imaging
Source: Research (Wash D C). 2023 Jul 19;6:0186. doi: 10.34133/research.0186 (PMC11740978; doi:10.34133/research.0186)
Supplement: Supplementary 1 — Supplementary Methods Tables S1 to S3 Figs. S1 to S27 [file research.0186.f1.docx]

Supporting Information

Imaging Carotid Plaque Burden in living mice via Hybrid Semiconducting Polymer Nanoparticles Based Near-Infrared-II Fluorescence and Magnetic Resonance Imaging

Li Xu, ^1 #^ **·** Zhe Li, ^1 #^ **·** Yuan Ma, ^1^ **·** Lingling Lei, ^1^ **·** Renye Yue, ^1^ **·** Hui Cao, ^1^ **·** Shuangyan Huan, ^1^ **·** Wei Sun, ^2, 3^ **·** Guosheng Song ^1 *^

**Supplementary Methods**

**Chemicals and materials.**

Main chemicals used in this experiment were purchased from Sigma Chemical Co. (St. Louis, MO, USA) and Aladdin Chemical Reagent Co. Ltd. (Shanghai, China). Compound 1 was synthesized according to previous literature ^[1]^. Three semiconducting polymers were obtained by multi-step reactions in organic solvents. The silica gel (200-300 mesh) used for column chromatography was purchased from Yantai Jiangyou Silica Gel Development Co., Ltd. DSPE-PEG_2000_-Cy5.5 was purchased from Ruixi (Xian, China). Fetal bovine serum (FBS), Dulbecco’s modified eagle medium (DMEM), RPMI-1640 medium and trypsin-EDTA solution (0.5% trypsin, 5.3 mM EDTA tetrasodium) were purchased from Adamas life. (Shanghai, China). Water was purified and doubly distilled by a Milli-Q system (Millipore, USA).

**Instrument and equipment**

UV-Visible absorption spectra were acquired via the Shimadzu UV-2600 UV-VIS-NIR spectrophotometer. Fluorescence spectra were recorded on a HITACHI F4600 fluorescence spectrophotometer with a 1 cm standard quartz cell. TEM images were acquired on a transmission electron microscope (Hitachi HT7700, Japan). Dynamic light scattering (DLS) measurements were conducted on a Nano ZS instrument (Malvern, UK). The hydrodynamic size was measured by Malvern Zetasizer Nano ZS90 (Malvern). ^1^H nuclear magnetic resonance (NMR) spectrum was performed on a Bruker DRX-400 spectrometer (Bruker) system. The fluorescent images of centrifuge tubes or mice were obtained via a near-infrared InGaAs array imaging system (Princeton, America) equipped with narrow-band interference-filter integrations. The NIR-II fluorescence images were analyzed by Image J 1.8.0 software. Fluorescent images of cells were obtained from the Olympus FV1000-MPE laser scanning confocal microscope (Japan).

**Synthesis of compound 2.**

The compound 1 (0.80 g, 0.62 mmol) was dissolved in 1,2-dichloroethane (20 mL) and transferred to a 100 mL three-necked flask and anhydrous N, N-dimethyl-formamide (DMF, 1.9 mL) was added under argon. Then, the mixture was transferred to an ice water bath, the POCl_3_ (1.0 mL) was added dropwise slowly and the reaction was stirred at 0 °C for 2 h. Next, the reaction was heated to 90 °C and stirred overnight. After the reaction, the mixture was extracted with DCM-H_2_O to remove DMF from the system. The organic layers were combined and purified with a silica gel column (eluent: DCM/ PE = 1/1, v/v). The compound 2 as orange-red viscous liquid was obtained (0.69 g, 63.73 % yield). 1H NMR (400 MHz, CDCl_3_) δH 10.15 (s, 2H), 4.76 (d, J = 7.2 Hz, 2H), 4.63 (d, J = 7.2 Hz, 4H), 3.21 (t, J = 7.6 Hz, 4H), 2.40 - 2.37 (m, 1H), 1.99 - 1.91 (m, 6H), 1.52 - 1.29 (m, 44H), 1.07 - 0.99 (m, 24H), 0.92 - 0.83 (m, 32H), 0.71 (t, J = 7.2 Hz, 12H) ^[1]^.

**Synthesis of compound M1.**

The compound 2 (0.50 g, 0.37 mmol) was dissolved in chloroform (30 mL) and transferred to a 100 mL three-necked flask. The compound 3 (0.30 g, 1.11 mmol) and pyridine (1 mL) was added under argon protection. The reaction was stirred at 65 °C overnight. After the reaction, the mixture was cooled to room temperature and poured into methanol to precipitate a blue solid. The mixture was filtrated and purified with a silica gel column (eluent: DCM/ PE = 1/1, v/v). The monomer M1 as a blue-black solid was obtained (0.50 g, 72.35% yield). 1H NMR (400 MHz, CDCl_3_) δH 9.18 (s, 2H), 8.85 (d, J = 1.2 Hz, 1H), 8.57 (d, J = 8.4 Hz, 1H), 8.02 (d, J = 1.6 Hz, 1H), 7.88 - 7.84 (m, 2H), 7.78 (d, J = 8.0 Hz, 1H), 4.74 - 4.72 (m, 6H), 3.23 (t, J = 7.6 Hz, 4H), 2.39 - 2.33 (m, 1H), 2.07 - 2.00 (m, 2H), 1.92 - 1.84 (m, 4H), 1.53 - 1.26 (m, 44H), 1.13 - 0.67 (m, 68H) ^[1]^.

**Synthesis of NIR-1，NIR-2 or NIR-3 polymer.**

The monomer of M1 (0.14 g, 0.08 mmol) and M2 (0.03 g, 0.08 mmol), M3 (0.07 g, 0.08 mmol) or M4 (0.05 g, 0.08 mmol) were dissolved with toluene (5 mL) in 50 mL two-necked flask, respectively. The catalyst of Pd(PPh_3_)_4_ (13 mg, 0.01 mmol) was quickly added to the flask under argon protection. Then, the mixture was heated to 110 °C and stirred for 24 hours. The reaction was cooled to room temperature and poured into methanol solution (200 mL) to precipitate a dark solid. Next, the solid was collected by filter paper and extracted with methanol, hexane, acetone and chloroform by Soxhlet extractor for 12 h, respectively. The extracting solution of chloroform was cooled to room temperature and concentrated by rotary evaporation. Finally, the solid was precipitated in methanol, filtered and dried to obtain dark solid NIR-1 (0.12 g, 70.59% yield), NIR-2 (0.15 g, 71.43% yield) or NIR-3 (0.14 g, 73.68% yield) ^[1]^.

**Calculation of Fluorescence Quantum Yield**

The quantum yield (QY) of NIR-1, NIR-2 and NIR-3 was determined in toluene solution, using IR26 of 0.05% as the reference. The QY was determined based on the following supporting equation (1):

$\boldsymbol{QY}_{\boldsymbol{s}}\boldsymbol{=}\boldsymbol{QY}_{\boldsymbol{r}}\boldsymbol{\times}\frac{\boldsymbol{A}_{\boldsymbol{r}}}{\boldsymbol{F}_{\boldsymbol{s}}}\boldsymbol{\times}{\boldsymbol{(}\frac{\boldsymbol{n}_{\boldsymbol{s}}}{\boldsymbol{n}_{\boldsymbol{r}}}\boldsymbol{)}}^{\boldsymbol{2}}$ **(1)**

where the **QY_s_** is the QY of NIR-1, NIR-2, or NIR-3, respectively, and **QY_r_** is the QY of IR26. **A** is the absorbance. **F** is the relative integrated fluorescence intensity in the region of 900 ~ 1500 nm, and **n** is the refractive index of the solvent.

**Synthesis of ultrasmall iron oxide nanoparticles**

Ultrasmall superparamagnetic iron oxide (SPIO) nanoparticles were synthesized using the pyrolysis method ^[2]^. 1.8 g iron-oleate and 3.22 g oleyl alcohol were dissolved in 10 g diphenyl ether, and then heated to 70 °C under vacuum for 1 h to remove oxygen and water. Next, the mixture was heated at 250 °C and kept for 30 min. After the heating source was removed, the colloid was stirred until the solution cool down to room temperature. The obtained iron oxide nanoparticles were washed with cyclohexane and precipitated by acetone.

**Preparation of SPNs(NIR-1), SPNs(NIR-2), SPNs(NIR-3), Cy5.5-labeled SPNs(NIR-1) or** **SPNs(NIR-1)@SPIO.**

All nanoparticles were prepared through the one-step nanoprecipitation method. A tetrahydrofuran (THF) solution (2 mL) containing NIR-1 (0.2 mg), or NIR-2 (0.2 mg), or NIR-3 (0.2 mg) and DSPE-PEG_2000_ (20 mg) was rapidly injected into distilled-deionized water (10 mL) under sonication. After sonication for another 10 min, the solution was evaporated at 50 °C by rotary evaporation to remove excess THF. Finally, the SPNs(NIR-1), SPNs(NIR-2) or SPNs(NIR-3) solution was purified by ultrafiltration (10 K, 6000 rpm) several times, respectively. The final concentration of SPNs was determined by the concentration of NIR-1, or NIR-2, or NIR-3.

For synthesis of Cy5.5-labeled SPNs(NIR-1), a THF solution (2 mL) containing NIR-1 (0.2 mg), DSPE-PEG_2000_-Cy5.5 (2 mg) and DSPE-PEG_2000_ (20 mg) were used. The other procedures were similar with that for prepration of SPNs(NIR-1).

For synthesis of superparamagnetic iron oxide hybrid nanoparticles (SPNs(NIR-1)@SPIO), a THF solution (2 mL) containing NIR-1 (0.2 mg), superparamagnetic iron oxide nanoparticles (0.2 mg), and DSPE-PEG_2000_ (20 mg) was used. The other procedures were similar with that for prepration of SPNs(NIR-1).

**NIR-II Fluorescence imaging in solution.**

The fluorescent images were acquired on a near-infrared InGaAs array imaging system (Princeton, America) under fluorescence mode with an acquisition time of 0.1 s and excitation wavelength at 808 nm (1 W/cm^2^). The fluorescent intensity in each image was quantified by applying a region of interest (ROI) over the image, using the image J, version 1.8.0.

For measuring NIR-II fluorescence intensity of SPNs(NIR-1), various concentrations of SPNs(NIR-1), SPNs(NIR-2), SPNs(NIR-3) or SPNs(NIR-1)@SPIO (e.g. 10 – 100 μg/mL) were irradiated by 808 nm laser for 500 ms.

For measuring fluorescence stability, SPNs(NIR-1) (10 μg/mL), SPNs(NIR-2) (10 μg/mL), SPNs(NIR-3) (10 μg/mL) and ICG (10 μg/mL) were irradiated by 808 nm laser for various time, respectively, followed by measuring NIR-II fluorescence intensity.

**Relaxation time and MRI images of** **SPNs(NIR-1)@SPIO**

The longitudinal and transversal relaxation time of SPNs(NIR-1)@SPIO were measured using a Bruker Minispec MQ60 NMR analyzer (Bruker, Germany), operating at a low magnetic field (1.4 T) and 37.0 ± 0.5 °C.

For collecting MRI images of SPNs(NIR-1)@SPIO, various concentrations of SPNs(NIR-1)@SPIO (200 μL, 0.1 - 0.7 mM Fe content) in tubes were scanned with a 7 T-MRI animal scanner (Pharma Scan 70/16 US, Burker), using T1 and T2 sequences, respectively.

**Colloidal stability of SPNs(NIR-1)**

For measuring fluorescence stability, SPNs(NIR-1) (10 μg/mL) were incubated in water, 1 × PBS buffer, 0.9% saline solution, and cell culture medium containing 10% FBS at room temperature. The size and size distribution of nanoparticles were determined by dynamic light scattering (DLS).

**Cellular culture.**

Macrophages (RAW264.7) or vascular endothelial cells (C166 cells) were cultured in DMEM containing 10% FBS and incubated at 37 °C and 5% CO2 in an incubator (Thermo Fisher Scientific, USA).

**In vitro** **cellular uptake**

For imaging cellular uptake, RAW264.7 cells were seeded in confocal dishes with a density of 1 × 10^5^ cells per well and cultured for 24 h, followed by treatment with Cy5.5-labeled SPNs(NIR-1) (50 μg/mL) for various time.

For imaging the endocytic difference between vascular epithelial cells and phagocytes *in vitro*, RAW264.7 and C166 cells were stimulated with LPS (0.1 μg/mL) for 24 h and were rinsed three times with PBS, and further incubated with Cy5.5-SPNs(NIR-1) (50 μg/mL) for 1.5 h. Then, those cells were rinsed three times with PBS.

The cells were imaged by fluorescent confocal imaging, using a laser scanning confocal microscope (Olympus FV1000-MPE, Japan).

**Cytotoxicity study**

To test the cytotoxicity, RAW264.7 cells were seeded into 96-well plates (2000 cells/well) and incubated with various concentrations of SPNs(NIR-1) for 24 h. Then, the cellular viability was tested by the standard MTT assay.

**Cellular experiment in vitro.**

Macrophages (RAW264.7) or vascular endothelial cells (C166 cells) were cultured in DMEM containing 10% FBS and incubated at 37 °C and 5% CO_2_ in an incubator (Thermo Fisher Scientific, USA).

For imaging endocytosis in macrophage in vitro, RAW264.7 cells were seeded in confocal dishes with a density of 1 × 10^5^ cells per well and cultured for 24 h, followed by treatment with Cy5.5-labeled SPNs(NIR-1) (50 μg/mL) for various time.

To study the endocytic difference between vascular epithelial cells and phagocytes *in vitro*, RAW264.7 cells and C166 were stimulated with LPS (0.1 μg/mL) for 24 h to establish a pro-inflammatory condition in vitro. Then, those LPS-treated cells were rinsed three times with PBS to remove the residual LPS, and further incubated with Cy5.5-SPNs(NIR-1) (50 μg/mL) for 1.5 h. Then, those cells were rinsed three times with PBS, followed by fluorescent confocal imaging, using a laser scanning confocal microscope (Olympus FV1000-MPE, Japan).

To test the cytotoxicity, RAW264.7 cells were seeded into 96-well plates (2000 cells/well) and incubated with various concentrations of SPNs(NIR-1) for 24 h. Then, the cellular viability was tested by the standard MTT assay.

**Atherosclerosis mice models**

All animal procedures were performed in accordance with the Guidelines for the Care and Use of Laboratory Animals of Hunan University.

Male C57BL/6 mice (8-week-old) were housed under controlled conditions (22 °C, 55 - 65% humidity, 12 h light-dark cycle) and were allowed free access to tap water. After one-week of acclimatization, the mice received treatments for different imaging purposes.

To prepare atherosclerosis mice (AS mice), C57BL/6 mice underwent the ligation of the left common carotid artery, while the no treatment of the right common carotid artery, followed by a high-fat diet (20 % fat and 1.5 % cholesterol) for 8 weeks. Under this condition, those animal models would develop to atherosclerotic lesions in the left carotid artery.^[3]^

To prepare atherosclerosis mice complicated with acute interstitial pneumonia mode (AS mice + AIP), LPS (2 mg/mL, 50 μL) was intranasally administered to induce pneumonia.^[4]^ Acute interstitial pneumonia injury would be established over the subsequent twenty-four hours. An infrared thermal imager (Teledyne FLIR TG165-X., USA) was used to record the body temperatures of healthy mice, AS mice and AS mice + AIP.

For neutrophile granulocyte counting and Giemsa staining of white blood cells, the venous blood samples collected from healthy mice, AS mice and AS mice + AIP into anticoagulant tubes, and tested by automatic hematology analyzer BC-2800vet (Mindray, Shanghai, China). About 10 μL of venous blood samples were smeared onto microscope slides, then air dried and stained with Giemsa (Applygen, Beijing, China). The slides were observed under an inverted fluorescence microscope (Zeiss, Model Axio Observer D1, Germany).

For measurement of blood lipid level, the venous blood samples of those mice in anticoagulant tubes were centrifuged at 3000 rpm/min, 4 °C for 15 min. Then, the supernatant was collected for measuring TG, TC, HDL-C, and LDL-C via automatic biochemical analyser Chemray 800 (Rayto, Shenzhen, China).

To prepare atherosclerosis mice complicated with intraplaque hemorrhage (AS mice + IPH), fresh arterial blood was collected and injected into the plaque lesion in left carotid artery of AS mice, via a microinjection needle to form small or large intraplaque hemorrhage.^[5]^ In detail, 5 μL of fresh arterial blood or 10 μL of fresh arterial blood was micro-injected to prepare AS mice + small IPH or AS mice + large IPH, respectively.

During operation, unnecessary bleeding should be avoided, and the anesthetized mice were warmed in a heater box to maintain body temperature.

This study was approved by the Institutional Animal Care and Use Committee of Hunan University. The approval/accreditation number: HUN-IACUC-2022-115.

**Pathological images of carotid arteries.**

The carotid artery was isolated for H&E staining to confirm the successful preparation of various atherosclerosis mice models. Both the left and right carotid arteries were carefully isolated after those mice were humanely executed by anesthetic. The separated carotid arteries were soaked in 4% paraformaldehyde fixative solution and fixed for 48 hours. Then, the arteries were completely embedded with OCT and frozen for sections. After gently rinsing the slides with water to remove water-soluble OCT, the sections were stained with hematoxylin and eosin. Finally, the sections were mounted by neutral balata and photographed by a digital histological slices scanner (Pannoramic MIDI, 3DHISTECH Ltd, Hungary).

For fluorescence confocal images of carotid arteries ex vivo, those sections of carotid arteries were incubated with 5% BSA blocking buffer (Applygen, P1625) for 30 min to block non-specific binding sites. Rat anti-mouse F4/80 antibody (1:200 in PBS, AbD Serotec, MCA497) and rabbit anti-mouse α-SMA antibody (1:200 in PBS, GeneTex, GTX629702) were applied to co-incubate the sections overnight at 4 °C. After PBS washing, Alexa Fluor 488-labeled goat anti-rat IgG (1:500 in PBS, CHAMOT, CM008-0.1A2F) and Alexa Fluor 594-labeled rabbit anti-mouse IgG (1:500 in PBS, CHAMOT, CM003-0.1A2F) were applied to co-incubate the sections for 2 h at room temperature. Following PBS washing, DAPI (Beyotime, P0131) was finally applied to stain the nucleus. The sections were observed by confocal microscope (Olympus FV1000-MPE, Japan).

**In vivo NIR-II fluorescence imaging of carotid atherosclerosis.**

The fluorescent images were acquired on a near-infrared InGaAs array imaging system (Princeton, America) under fluorescence mode using 1040 nm long-pass filter with a total acquisition time of 3h and excitation laser wavelength at 808 nm (1 W/cm^2^). The fluorescent intensity in each image was quantified by applying a region of interest (ROI) over the image, using the image J, version 1.8.0.

Detersile mice were performed with whole-body NIR-II fluorescence imaging at 30 min post *i.v.* injection of SPNs(NIR-1) (200 μg/mL, 200 μL). Vascular fluorescence signals of mice from torso and limb were adequately collected.

For NIR-II imaging unstable plaque *in vivo*, healthy mice, AS mice and AS mice + AIP were *i.v.* injected with SPNs(NIR-1) (200 μg/mL, 200 μL). After injection, those mice were performed with NIR-II fluorescence imaging of the upper extremities. At 6 hours post *i.v.* injection, the carotid arteries were isolated for NIR-II imaging *ex vivo*.

For NIR-II imaging intraplaque hemorrhage, AS mice, AS mice + small IPH, AS mice + large IPH were *i.v.* injected with SPNs(NIR-1) (200 μg/mL, 200 μL) at 1 h post microinjection of fresh arterial blood. Immediately, those mice were performed with NIR-II fluorescence imaging of the cervical region. At 6 hours post *i.v.* injection, the carotid arteries were isolated for NIR-II imaging *ex vivo*.

Seven days after imaging, the main organs of those mice were collected for H&E staining via a standard protocol.

**In vivo MRI imaging of carotid atherosclerosis.**

Healthy mice and AS mice were scanned with a 7 T-MRI animal scanner (Pharma Scan 70/16 US, Burker) via using T_2_-weitght MRI sequence, respectively, before injection. Then, healthy mice and AS mice were *i.v.* injected with SPNs(NIR-1)@SPIO (200 μg/mL, 200 μL). Twenty-four hours post *i.v.* injection, those mice were scanned using T_2_-weitght MRI sequence, respectively, as following parameters:

Slice thickness = 0.7 mm; FOV = 30 × 30 cm;

T_2_WI MRI images: TR/TE = 2200/ 8 ms;

T_2_- relaxation times were calculated with the Paravision 360 software.

The DICOM data of tomographic images exported from the MRI system was analyzed by imaging software. The T_2_-weighted signal changes of atherosclerotic plaque or clear lumen in left carotid artery were calculated at pre- and 24 h post-injection, respectively.

**Statistical analysis.**

The statistical analysis was shown as the mean ± standard deviation (SD) of the mean. Statistical significance (*P< 0.05, **P< 0.01, ***P< 0.001) was executed via one-way analysis of variance (ANOVA).

**Supplementary Tables**

Table S1. The representative advanced studies of fluorescence probes for imaging carotid atherosclerosis.

| **Probes** | **Excitation (nm)**  **/Emission (nm)** | **Size (nm)** | **In vivo**  **/Ex vivo** | **Samples** | **Ref.** |
| --- | --- | --- | --- | --- | --- |
| Cy5.5-LyP-Hsp | 678/689 | 12.9 | In vivo  /Ex vivo | ApoE^-/-^ mice | ^[6]^ |
| Cy5.5-OPN-DMSA-MNPs | - | 7.3 ± 0.6 | In vivo  /Ex vivo | ApoE^-/-^ mice | ^[7]^ |
| ICG | 775/845 | - | Ex vivo | Human specimen | ^[8]^ |
| Hemoglobin | 630/650 | - | Ex vivo | Human specimen | ^[9]^ |
| Bilirubin | 785/794 - 1144 | - | Ex vivo | Human specimen | ^[10]^ |
| DS-Ce6 | 640/720 | 50.1 ± 1.1 | In vivo  /Ex vivo | ApoE^-/-^ mice | ^[11]^ |
| CLIO-AF750 | 546/90 | 35 | Ex vivo | ApoE^-/-^ mice | ^[12]^ |
| IRPNPs | 776 | 285.8 | In vivo | C57BL/6 mice | ^[13]^ |
| CSNP | 480/530 | 100 | Ex vivo | ApoE^-/-^ mice | ^[14]^ |
| UCNP-anti-OPN probe | 980/542, 656 | 20.5 ± 0.8 | In vivo | ApoE^-/-^ mice | ^[15]^ |
| SPNs(NIR-1)@SPIO | 808/900 - 1100 | 50 ~ 58 | In vivo  /Ex vivo | C57BL/6 mice | This work |

Table S2. Synthesis of NIR-1, NIR-2 and NIR-3.

| **Substrate material (g)** | **Substrate material (g)** | **Main products (g)** | **Yield (%)** |
| --- | --- | --- | --- |
| M1 (0.14) | M2 (0.03) | NIR-1 (0.12) | 70.59 |
| M1 (0.14) | M3 (0.07) | NIR-2 (0.15) | 71.43 |
| M1 (0.14) | M4 (0.05) | NIR-3 (0.14) | 73.68 |

Table S3. Summary of the Spectral Data of Dyes in toluene.

| **Monomer dyes** | **λ_max_^abs^(nm)*^a^*** | **λ_max_^em^(nm)*^b^*** | **Ф (%)*^c^*** |
| --- | --- | --- | --- |
| **NIR-1** | 790 | 862 | 3.31% |
| **NIR-2** | 800 | 857 | 2.16% |
| **NIR-3** | 810 | 852 | 2.29% |

* ***a.*** Absorption maximum. ***b.*** Emission maximum. ***c.*** Fluorescnece Quantum yield.

**Supplementary Figures**

Figure S1. Synthesis of NIR-1, NIR-2, and NIR-3.


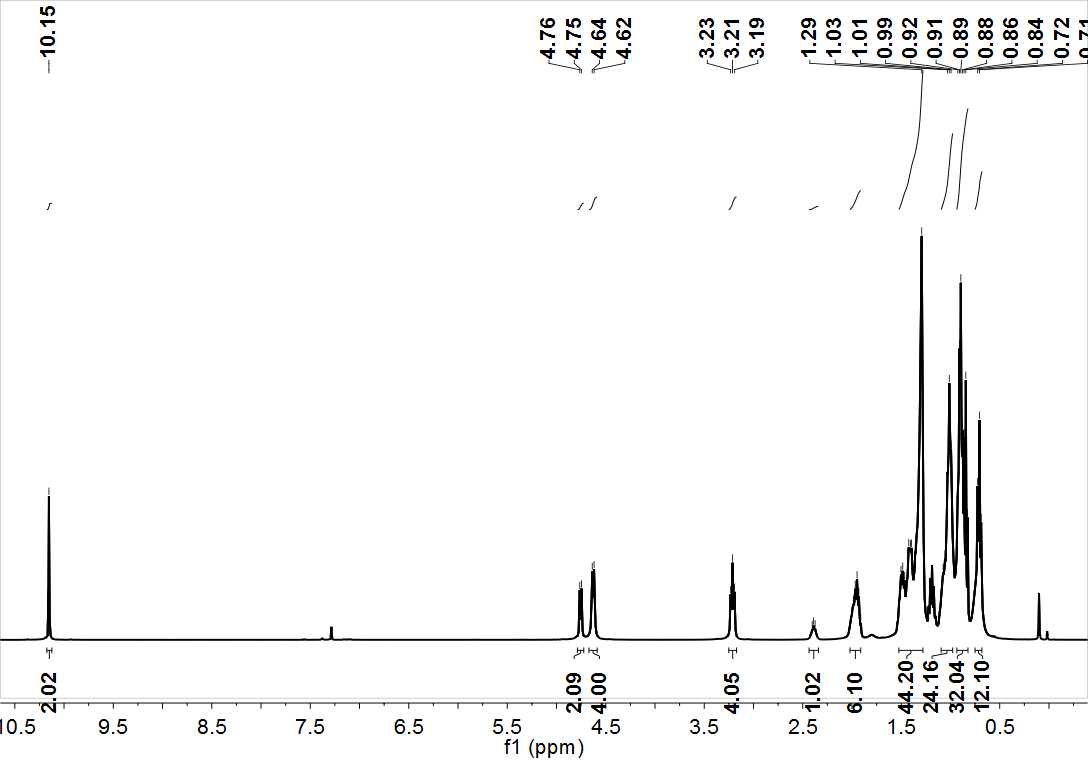


Figure S2. ^1^H NMR spectrum of compound 2.


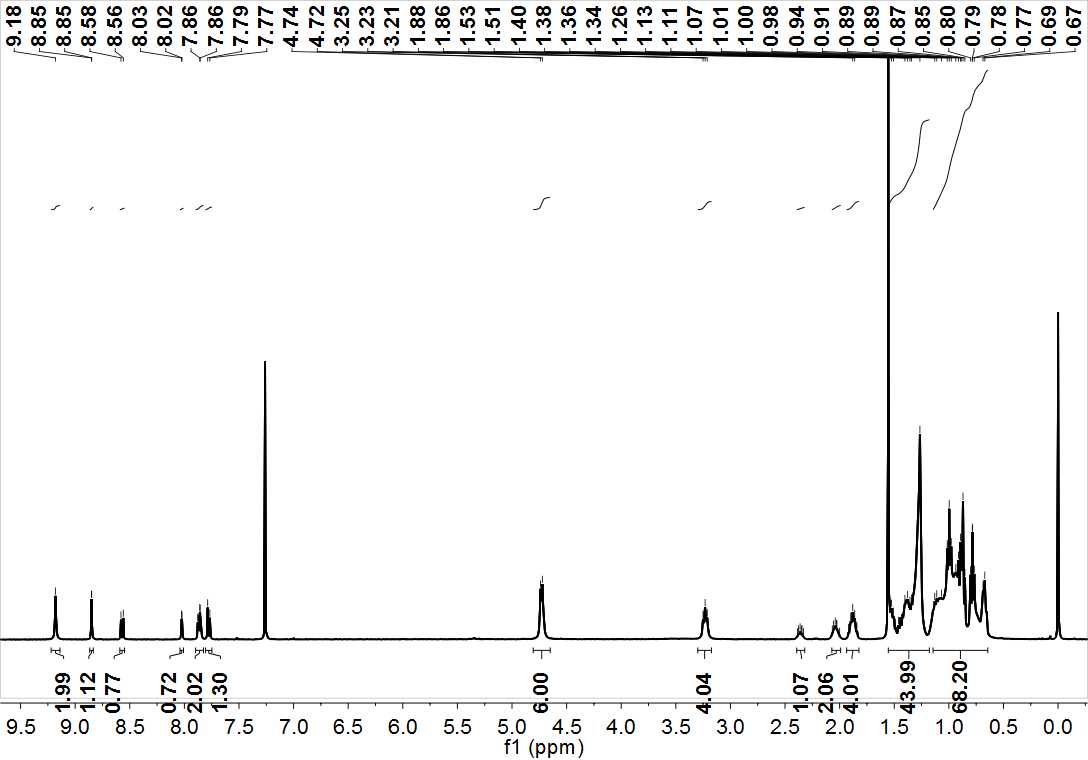


Figure S3. ^1^H NMR spectrum of M1.


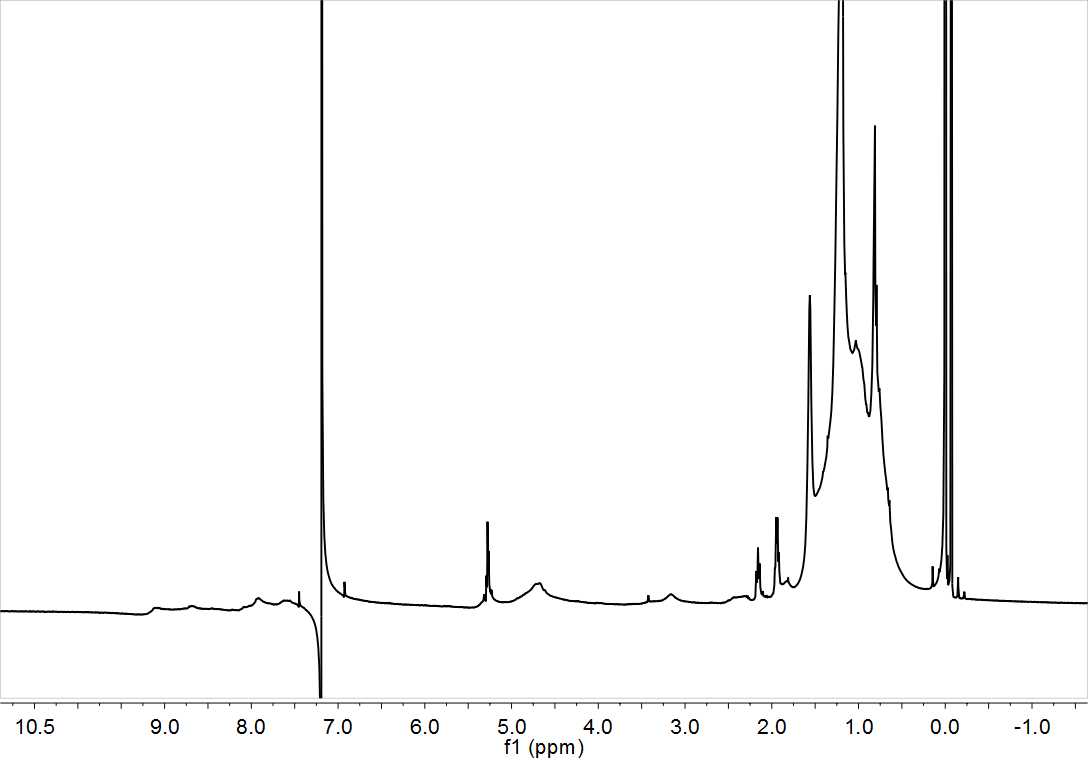


Figure S4. ^1^H NMR spectrum of NIR-1.


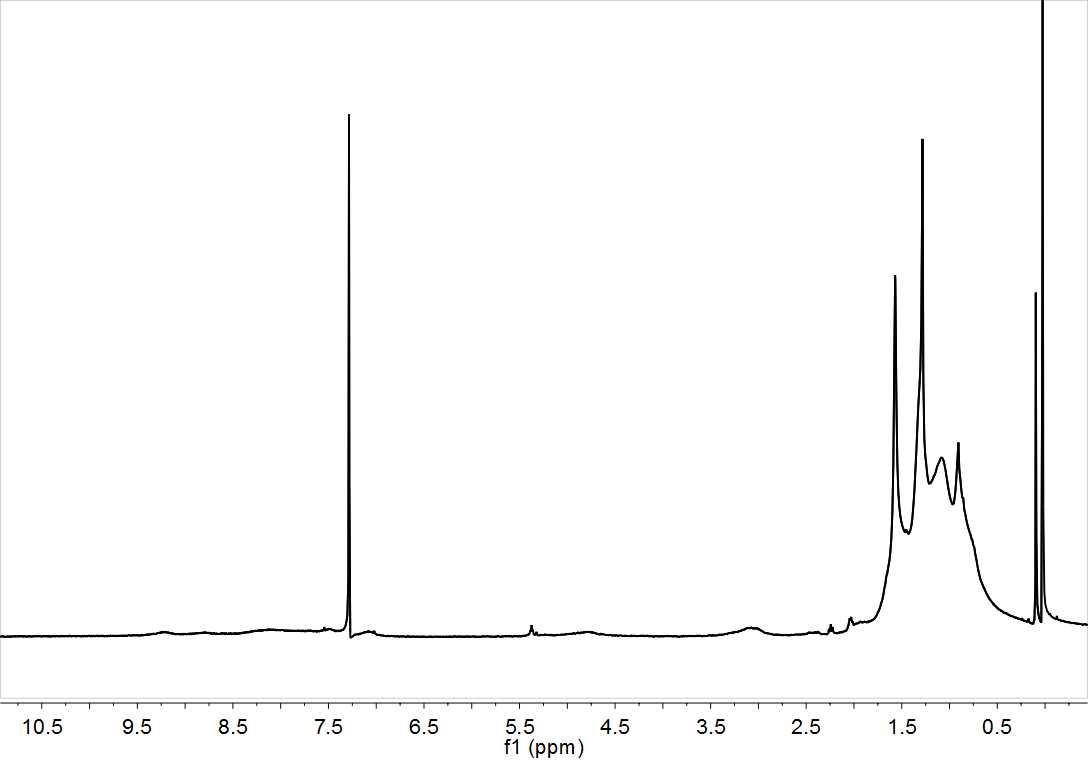


Figure S5. ^1^H NMR spectrum of NIR-2.


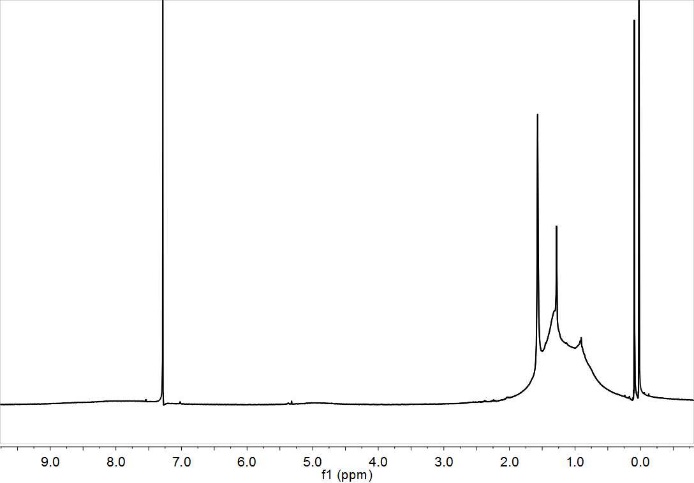


Figure S6. ^1^H NMR spectrum of NIR-3.

Figure S7. The schematic diagram for preparation of semiconducting polymer nanoparticles SPNs(NIR-1) using DSPE-PEG_2000_, via nano-precipitation method.

Figure S8. DLS size distribution of SPNs(NIR-1).

Figure S9. Colloidal stability of SPNs(NIR-1) within 21 days in water, 1 × PBS buffer, 0.9% saline solution, and cell culture medium containing 10% FBS.

Figure S10. The concentration-dependent fluorescence intensity of SPNs(NIR-1).

Figure S11. Fluorescence intensity of SPNs(NIR-1), SPNs(NIR-2), SPNs(NIR-3) and ICG (10 μg/mL) under 808 nm laser irradiation (1 W/cm^2^) for different time.

Figure S12. Representative TEM image of ultrasmall superparamagnetic iron oxide nanoparticles (SPIO).

Figure S13. DLS size distribution of SPNs(NIR-1)@SPIO.

Figure S14. The (a) T_1_- and (b) T_2_- weighted magnetic resonance imaging (MRI) signal intensity of SPNs(NIR-1)@SPIO, calculated from Figure 1o.

Figure S15. NIR-II fluorescence intensity of SPNs(NIR-1)@SPIO with different Fe concentrations (mM), calculated from Figure 1p.

Figure S16. Whole-body NIR-II fluorescence image for C57BL/6 mice post *i.v.* injected with SPNs(NIR-1).

Figure S17. NIR-II fluorescence images of saphenous vein and femoral artery for C57BL/6 mice *i.v.* injected with SPNs(NIR-1). (a) Two-dimensional and (b) Three-dimensional NIR-II fluorescence imaging of saphenous vein. (c) The fluorescence intensity profiles (dots) along the orange dashed line in saphenous vein (a). (d) Two-dimensional and (e) Three-dimensional NIR-II fluorescence imaging of femoral artery. (f) The fluorescence intensity profiles (dots) along the orange dashed line in femoral artery (d).

Figure S18. Three-dimensional NIR-II fluorescence imaging of carotid arteries of C57BL/6 mice post *i.v.* injected with SPNs(NIR-1), drawn from Figure 2c.

Figure S19. Prussian blue iron stained the right (a) and left (b) carotid arteries section, and the quantification of blue granules per view (c) (20X, bar = 50 μm).

Figure S20. Plaque area% (%) of healthy mice, AS mice and AS mice + AIP.

Figure S21. Representative Giemsa staining of blood smear of healthy mice, AS mice and AS mice + AIP (Bar = 100 μm).

Figure S22. Lung Wet/Dry ratio of healthy mice, AS mice and AS+AIP mice. P-values were determined using one-way ANOVA: *P< 0.05.

Figure S23. Erythrocyte sedimentation rate (a), C-reactive protein (b), and procalcitonin (c) of healthy mice, AS mice and AS mice + AIP.

Figure S24. The quantified fluorescence intensity of right carotid arteries (RCA) at different time points for healthy mice, AS mice and AS mice + AIP groups.

Figure S25. Representative photos and fluorescence images of neck regional anatomy of healthy mice (a) and AS mice (b). Green arrow or dot line: gland, blue arrow: carotid vein, yellow arrow: trachea, red arrow: left carotid artery (LCA). Bar = 5 mm. MRI image size was 40 × 40 mm^2^. (H = head, F = feet, L = left, R = right)


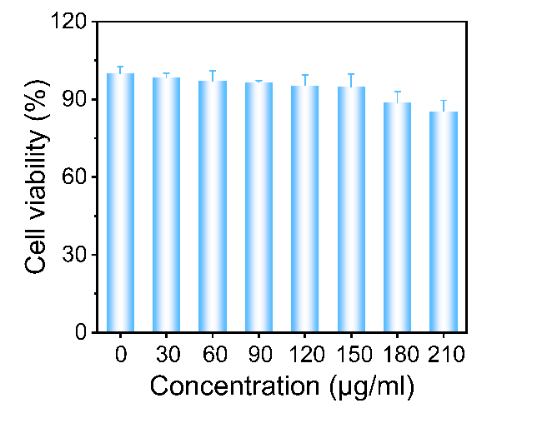


Figure S26. The relative cell viability of SPNs(NIR-1).

Figure S27. The toxicity of SPNs(NIR-1) towards major organs. Representative H&E staining of the major organs (lung, liver, spleen, kidney, and heart) from healthy mice, AS mice, AS mice + AIP, AS mice + small IPH, and AS mice + large IPH at 7 d post intravenous injection of SPNs(NIR-1). (Bar = 100μm).

## SI Reference

[1] Z. Li, H. g. Chen, J. Yuan, J. Zou, J. Li, H. l. Guan, Y. p. Zou, *J. Cent. South Univ.* **2021**, 28, 1919.

[2] R. Yue, C. Zhang, L. Xu, Y. Wang, G. Guan, L. Lei, X. Zhang, G. Song, *Chem* **2022**, 8, 1956.

[3] W. Chen, S. Tumanov, S. M. Y. Kong, D. Cheng, E. Michaelsson, A. Bongers, C. Power, A. Ayer, R. Stocker, *Redox Biol.* **2022**, 58, 102532.

[4] Y. Ma, L. Xu, B. Yin, J. Shang, F. Chen, J. Xu, Z. L. Song, B. Nan, G. Song, X. B. Zhang, *Nano Lett.* **2021**, 21, 4484.

[5] D. Pan, W. Wu, G. Zuo, X. Xie, H. Li, X. Ren, C. Kong, W. Zhou, Z. Zhang, M. Waterfall, S. Chen, *Cell. Signal.* **2022**, 98, 110419.

[6] M. Uchida, H. Kosuge, M. Terashima, D. A. Willits, L. O. Liepold, M. J. Young, M. V. McConnell, T. Douglas, *ACS Nano* **2011**, 5, 2493.

[7] H. Qiao, Y. Wang, R. Zhang, Q. Gao, X. Liang, L. Gao, Z. Jiang, R. Qiao, D. Han, Y. Zhang, Y. Qiu, J. Tian, M. Gao, F. Cao, *Biomaterials* **2017**, 112, 336.

[8] J. W. Verjans, E. A. Osborn, G. J. Ughi, M. A. Calfon Press, E. Hamidi, A. P. Antoniadis, M. I. Papafaklis, M. F. Conrad, P. Libby, P. H. Stone, R. P. Cambria, G. J. Tearney, F. A. Jaffer, *J. Am. Coll. Cardiol.* **2016**, 9, 1087.

[9] M. S. Albaghdadi, R. Ikegami, M. B. Kassab, J. A. Gardecki, M. Kunio, M. M. Chowdhury, R. Khamis, P. Libby, G. J. Tearney, F. A. Jaffer, *Arterioscler. Thromb. Vasc. Biol.* **2021**, 41, e385.

[10] N. M. Htun, Y. C. Chen, B. Lim, T. Schiller, G. J. Maghzal, A. L. Huang, K. D. Elgass, J. Rivera, H. G. Schneider, B. R. Wood, R. Stocker, K. Peter, *Nat. Commun.* **2017**, 8, 75.

[11] J. W. Song, J. W. Ahn, M. W. Lee, H. J. Kim, D. O. Kang, R. H. Kim, U. G. Kang, Y. H. Kim, J. Han, Y. H. Park, H. S. Nam, H. Yoo, K. Park, J. W. Kim, *J. Nanobiotech.* **2021**, 19, 338.

[12] J. R. McCarthy, E. Korngold, R. Weissleder, F. A. Jaffer, *Small* **2010**, 6, 2041.

[13] F. Chen, J. Chen, C. Han, Z. Yang, T. Deng, Y. Zhao, T. Zheng, X. Gan, C. Yu, *J. Mater. Chem. B* **2021**, 9, 4134.

[14] H. Kim, S. Kumar, D. W. Kang, H. Jo, J. H. Park, *ACS Nano* **2020**, 14, 6519.

[15] R. Qiao, H. Qiao, Y. Zhang, Y. Wang, C. Chi, J. Tian, L. Zhang, F. Cao, M. Gao, *ACS Nano* **2017**, 11, 1816.
